# Are Sensory TRP Channels Biological Alarms for Lipid Peroxidation?

**DOI:** 10.3390/ijms150916430

**Published:** 2014-09-17

**Authors:** Seung-In Choi, Sungjae Yoo, Ji Yeon Lim, Sun Wook Hwang

**Affiliations:** Department of Biomedical Sciences and Department of Physiology, Korea University College of Medicine, Seoul 136-705, Korea; E-Mails: csiat@korea.ac.kr (S.-I.C.); headyoo@korea.ac.kr (S.Y.); ljyangel1004@korea.ac.kr (J.Y.L.)

**Keywords:** sensory TRP ion channels, pain, lipid peroxidation, oxidative stress

## Abstract

Oxidative stress induces numerous biological problems. Lipid oxidation and peroxidation appear to be important steps by which exposure to oxidative stress leads the body to a disease state. For its protection, the body has evolved to respond to and eliminate peroxidation products through the acquisition of binding proteins, reducing and conjugating enzymes, and excretion systems. During the past decade, researchers have identified a group of ion channel molecules that are activated by oxidized lipids: transient receptor potential (TRP) channels expressed in sensory neurons. These ion channels are fundamentally detectors and signal converters for body-damaging environments such as heat and cold temperatures, mechanical attacks, and potentially toxic substances. When messages initiated by TRP activation arrive at the brain, we perceive pain, which results in our preparing defensive responses. Excessive activation of the sensory neuronal TRP channels upon prolonged stimulations sometimes deteriorates the inflammatory state of damaged tissues by promoting neuropeptide release from expresser neurons. These same paradigms may also work for pathologic changes in the internal lipid environment upon exposure to oxidative stress. Here, we provide an overview of the role of TRP channels and oxidized lipid connections during abnormally increased oxidative signaling, and consider the sensory mechanism of TRP detection as an alert system.

## 1. Introduction

Oxidative stress caused by excessive oxygen exposure or imbalance of oxygen turnover may result in a disease state by hampering normal tissue functions. Reactive oxygen species (ROS) are the initiator components for this aberrant action. Mounting evidence indicates that reactive carbon and nitrogen species, which are formed from ROS metabolism, propagate and amplify pathologic signaling cascades. Peroxidized lipids are considered to comprise the majority of carbon species. Indeed, the detrimental influence of lipid peroxidation (LPO) products on molecules involved in essential signal transduction pathways continue to be identified in atherosclerosis, diabetes, and neurodegenerative diseases, as well as various types of inflammation. Accordingly, the detailed molecular nature of pathologic cascades, enzymatic tissue protective mechanisms, and sensory detection of LPO states are drawing significant attention [1]. In this review, we focus on neurosensory components responsible for detecting increased levels of the pathologic lipids and generating electrical signals toward the nervous system in response.

At the forefront of the somatosensory and visceral sensory pathway, a group of transient receptor potential (TRP) ion channels serves a central role for detecting of environmental changes [2]. These sensory TRP channels are expressed in the terminals of primary afferent nerve fibers and detect mechanical, thermal, and chemical stimuli. Upon detection, TRP channels open their pores, whereby cation influx depolarizes the nerve fibers. Perception of a stimulus occurs if the electrical signal generated by TRPs reaches a given threshold for exciting the nerve fibers and the nerve firing is transmitted to the higher brain regions.

Surprisingly, accumulating information about the identities of their chemical stimulators and their categorization suggests that those include many of LPO products [3]. TRPA1 and TRPV1, namely pain-mediating TRP channels, have been studied extensively in pathological lipid interactions. Here, we briefly introduce the sensory roles for these two TRPs first, discuss their interactions with LPO product, and speculate on the possible biological meanings of these interactions and unanswered questions.

## 2. Major Nociceptive TRPs: TRPA1 and TRPV1

Sensory receptors exist in the primary afferent termini, which innervate skin dermis/epidermis and visceral epithelia. Located in the surface of the nerve termini, those receptors sense external and internal environmental changes such as temperatures, mechanical stretches, and presence of exogenous or endogenous chemicals. In particular, extreme environments that potentially damage tissues including noxious cold or hot temperatures, intense mechanical contact, chemical toxins, pollutants, and internal pro-inflammatory mediators are detected by sensory receptors in the so-called pain fibers, a subset of sensory afferents, known as C-fibers and Aδ-fibers. The major nociceptive sensory receptors in these nerve fibers are TRPA1 and TRPV1 ion channel proteins [3,4]. They serve as transducers for a diverse array of harmful signals via allosteric pore opening (channel activation) to produce electrical signals. The electrical signals (depolarization) are due to cation influx through the activated TRP channel. The signals are amplified and propagated via activations of voltage gated cation channels, which is a natural property of excitable nerve fibers. The current set of nociceptive modalities in charge of TRPA1 and TRPV1 are introduced briefly below.

### 2.1. TRPA1

**Figure 1:**
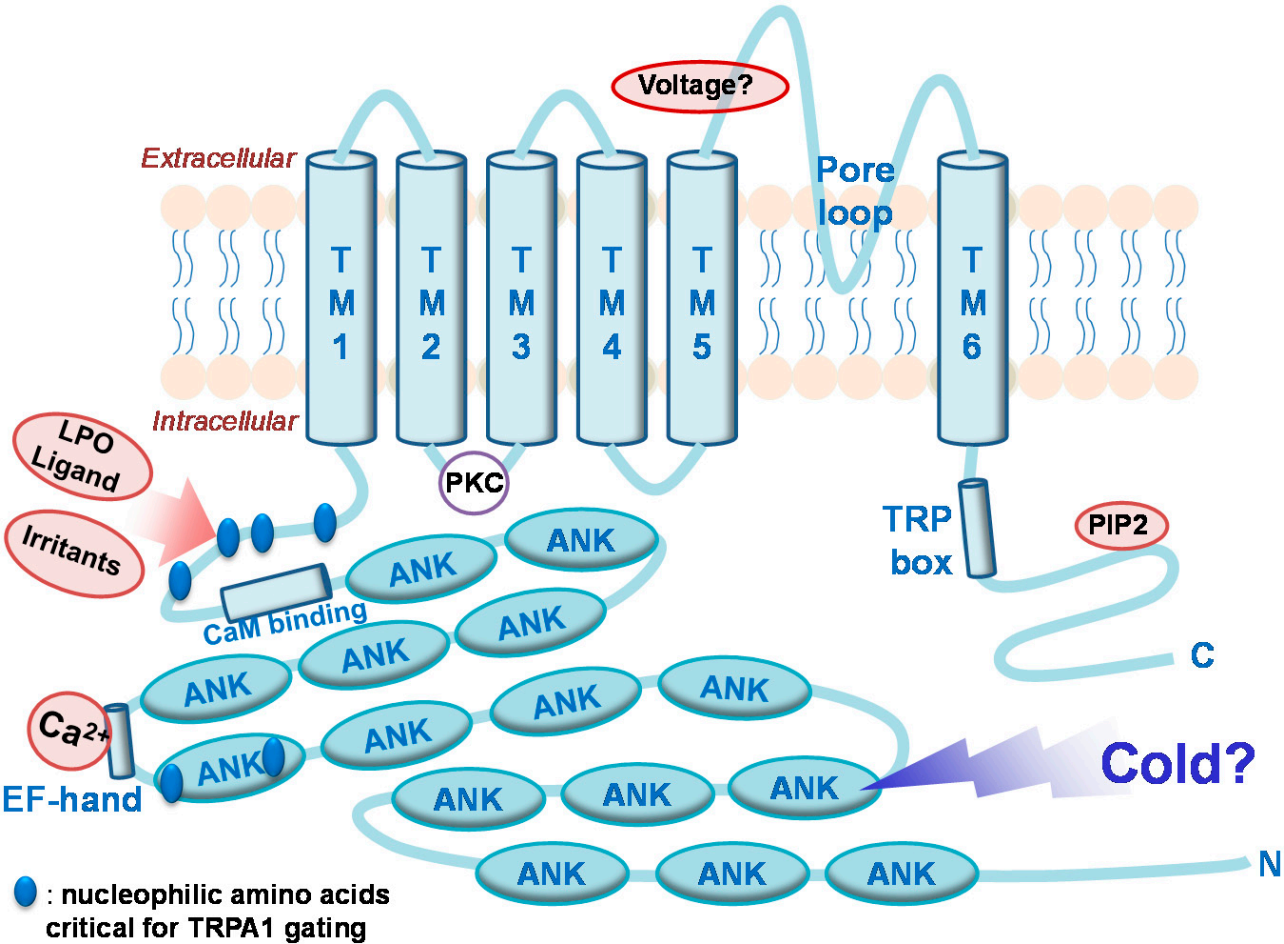
Transient receptor potential (TRP)A1 topology highlighting key domains, phosphorylation sites, and ligand binding pockets that affect gating functions. Abbreviations: TM, transmembrane domain; LPO, lipid peroxidation; ANK, ankyrin repeat; CaM binding, calmodulin binding site; PIP2, phosphatidylinositol 4,5-bisphosphate; PKC, substrate regions for protein kinase C actions; TRP box, conserved amino acids in TRPA, TRPC, TRPV, and TRPM subfamilies; Voltage, predicted voltage-sensing domain.

Containing a relatively large number of ankyrin repeats (~15) compared with other TRP channels, this subtype is named an ankyrin subtype [5] (Figure 1). The helical shape of these long repeats may confer stretchable properties to enable mechanosensitivity. In addition, certain specific amino acids within the ankyrin repeats have been thought to be crucial for detection of temperature changes and others to be related to chemical binding [6,7,8] (Figure 1). While a number of animal species have multiple ankyrin subtype paralogs (e.g., fruit fly, nematode, and zebra fish), mammals—Including humans—Only express TRPA1. Owing to its polymodal properties to detect heterogeneous harmful signals, which are discussed in this review, TRPA1 is often referred to as a “damage-sensing channel” and is recognized as an important target for inhibiting pain. TRPA1 was first cloned in an oncology study [9], and was later rediscovered as a sensor for painful cold temperatures (<17 °C) [10]. Although the cold sensitivity of the human TRPA1 ortholog remains a matter of debate [11], the cold sensing function of TRPA1 in murine and rodent species has been confirmed several times during the last decade [11,12,13,14,15,16,17]. According to the same animal studies, TRPA1 seems to play a central role in painful cold hypersensitivity in the context of injury or inflammation-related pathological pain. In addition, TRPA1 participates, at least in part, to the sensation of painful mechanical stretching [15,16,18].

Not only physical (thermal and mechanical) sensitivities, but also TRPA1 has been shown to exhibit chemical sensitivity to multiple damaging substances, and thus it is considered to be highly polymodal. Even the chemical pool for TRPA1 activators continues to grow [3,19,20,21]. Interestingly, reactive compounds with α,β-unsaturated carbonyl moieties, which can potentially bind to nucleophilic amino acids (lysine, serine, histidine, and cysteine) on TRPA1 *N*-terminal ankyrin repeats in a covalent binding manner, constitute the majority of TRPA1 activators. During the early stage of the TRPA1 pharmacological studies, most of the activating molecules were identified from popular natural ingredients such as mustard oil, cinnamon oil, and garlic oil [5,22,23,24], all of which are later known to follow the covalent binding mechanism. LPO products that activate TRPA1, including 8-iso prostaglandins (PGs) and related cyclopentenone PGs also fall into this category (see below). In addition, environmental pollutants including formaldehyde, acetaldehyde, industrial isothiocyanate, hypochlorite, and acrolein (which is also produced during endogenous LPO processes), have been shown to cause irritation, coughing, and pain by stimulating TRPA1 through the same covalent binding mechanism [6,7,25,26,27,28,29].

Many pathologic lipids endogenously generated under inflammation or oxidative stress are also TRPA1 activators, some of which follow the covalent binding mechanism. Prime examples include nitro-fatty acids and P450 epoxygenase products (epoxyeicosatrienoic acids: EETs). Increased during inflammation, tissue nitric oxide (NO) often binds to membrane lipids to form nitrated phospholipids and fatty acids [30]. Again, nitro-fatty acids activate TRPA1 by covalently binding its nitrated carbon to TRPA1 protein. Among endogenously-released TRPA1 activating substances, including the LPO metabolites described below, nitro-oleic acid, a form of nitro-fatty acid, exhibits one of the highest potencies (EC50 of 1 µM). Although NO itself was once shown to be a weak activator of TRPA1 and TRPV1 [31], the possible dissociation of NO from nitro-oleic acid does not seem to mediate the same effect of nitro-oleic acid on TRPA1. This is because nitro-oleic acid is more potent than known NO donors, and NO scavengers do not protect from TRPA1 activation [32]. However, TRPA1 specificity of nitro-oleic acid has been questioned since nitro-oleic acid has recently been shown to activate TRPV1 as well as other as-yet to be identified ion channels [33,34]. Inside neurons on the sensory afferent pathways (trigeminal and dorsal root ganglionic (DRG) neurons, and the dorsal horn neurons), P450 epoxygenase is known to produce EETs [35,36]. Interestingly, TRPA1 is also present in the central termini of DRG neurons and helps synaptic functions to relay mechanical allodynia signaling. EETs appear to facilitate spontaneous excitatory postsynaptic currents (sEPSCs) via activation of TRPA1 by covalent binding with *N*-terminal cysteine/lysine residues [36]. It remains to be clarified which locations among pre- or post-synapses, or supporting cell types are the most important for EET generation and release.

Another centrally acting, but probably non-covalently bound lipid species was found: hepoxilins. Hepoxilins are generated by neighboring cells and contribute to hyperalgesic synaptic strengthening by activating TRPA1 and TRPV1 at the central C-fiber terminals [37]. It is interesting that a parent molecule of hepoxilin biosynthesis, 12(*S*)-HpETE is a TRPV1 activator (see below). Second messenger lipids that open TRPA1 channels upon G protein-coupled receptor activation seem to follow a typical non-covalent binding mode. In response to the inflammatory mediator bradykinin, B2 receptor activation triggers Gq protein signaling, which itself generates arachidonic acid and diacylglycerol (DAG) via phospholipase A2 (PLA2) and phospholipase C (PLC) activation, respectively. Both arachidonic acid and DAG activate TRPA1 [5]. While arachidonic acid activation appears to occur via a direct mode, DAG may require further degradation into polyunsaturated fatty acids (PUFAs) including arachidonic acid in order to bind TRPA1. In addition, DAG itself may lead to TRPA1 phosphoryation by activating kinases, which in turn may facilitate TRPA1 activation. Collectively, TRPA1 is a polymodal detector for diverse harmful insults.

An interesting feature rarely found in other TRP channels is species difference in ligand pharmacology. For example, menthol shows bimodal actions on mouse TRPA1 by activating it at lower concentrations and inhibiting it at higher concentrations whereas menthol only activates human TRPA1 [38,39]. Caffeine activates mouse TRPA1 channels but inhibits human TRPA1 [40]. Similar pharmacological discrepancy has also appeared in the developmental processes of the industry searching for synthetic TRPA1 modulators [41,42]. This seems to be caused by low sequence identity among TRPA1 orthologs: human and murine TRPA1 are only 80% identical, which is relatively low for sensory TRP channels (commonly, 87%–95%). The evolutionary pressures for the low similarity and resultant varied sensitivities of TRPA1 remain unexplored.

### 2.2. TRPV1

**Figure 2:**
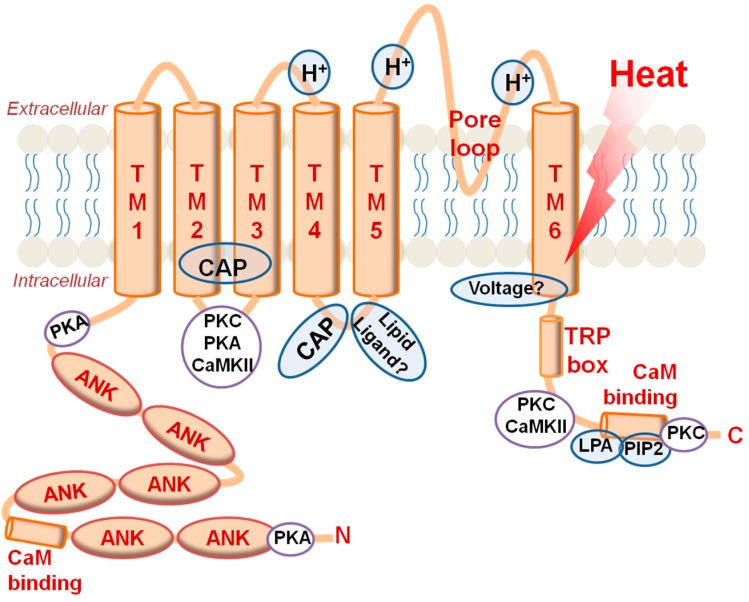
TRPV1 topology highlighting key domains, phosphorylation sites, and ligand binding pockets that affect gating functions. Abbreviations: H^+^, proton binding site; TM, transmembrane domain; CAP, capsaicin binding region; PKC, substrate regions for protein kinase C actions; PKA, substrate regions for protein kinase A actions; CaMKII, substrate regions for Ca^2+^/calmodulin-dependent protein kinase action; Voltage, predicted voltage-sensing domain; TRP box, conserved amino acids in TRPA, TRPC, TRPV, and TRPM subfamilies; CaM binding, calmodulin binding site; LPA, lysophosphatidic acid binding site; PIP2, phosphatidylinositol 4,5-bisphosphate.

The identification of TRPC (canonical subtype) in drosophila and mammals prompted quests for other TRP subtypes [43,44,45], which resulted in the identification of TRPV1 as the first non-canonical member of the TRP family [46]. With respect to its somatosensory and nociceptive functions, TRPV1 is also the first sensory TRP channel identified (Figure 2). TRPV1 may cover the most central and broadest sensory modalities. It is named after the famous natural vanilloid, capsaicin, which is present in red peppers and is a specific TRPV1 activator. TRPV1 is not only activated by the pungent vanilloids, but also by noxiously high temperatures (>42 °C), hyperosmolalities, acidic pH (protons), a variety of lipidergic irritating chemicals, and polyamines [46,47,48,49,50], which indicates that TRPV1 is a polymodal detector sensitive to thermal, mechanical, and chemical insults.

The body of evidence for TRPA1 polymodality is comparable to TRPV1; however, expression of TRPV1 is more readily observed in C- and Aδ-nociceptors. Furthermore, other TRPV cousins such as TRPV2–V4 also serve important roles for different types of modalities in the periphery [3,4]. Therefore, TRPV1 appears to be a more universal sensory molecule than TRPA1, and TRPVs are evolutionarily differentiated for covering a diverse array of environmental changes. Interestingly, TRPA1 expression is only detected in a subset of TRPV1-expressing C-fibers [10]. Thus, it is possible that TRPV1 may first define the pain mediating role for a specific subset of sensory fibers (especially unmyelinated C- and thinly myelinated Aδ-fibers) and that, among a pre-defined nociceptive subset, a smaller population may acquire an additional capacity for coping with an extended list of harmful conditions. Another possibility is that TRPV1 may have been naturally selected as a general alarm against dangers that more frequently occur in competition with TRPA1. In any case, TRPV1/TRPA1-double positive C-fibers are referred to as polymodal nociceptors because they can sense an extremely extensive number of damaging signals. Such polymodal nociceptors are found not only in somatosensory nerves but also in the visceral nerves [51,52].

**Figure 3:**
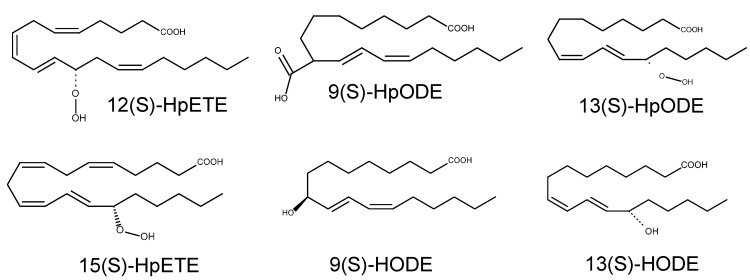
Lipid hydroperoxides (LOX metabolites).

The first examples of specific TRPV1 activators in endogenous substances were identified in amine conjugate studies. The structural similarity between amine conjugates and capsaicin (a carbon chain—An amide bond—a catecholic or guaiacolic aromatic ring) may lead to the idea that those substances share the same TRPV1 binding pocket. Such amine conjugates include anandamide, *N*-arachidonoyl dopamine (NADA), *N*-oleoyl dopamine (OLDA), and *N*-oleoyl ethanolamine (OEA) [53,54,55,56,57]. Anandamide, which is generated in C-fibers, was recently demonstrated to amplify nociceptive signals by activating TRPV1 [58,59]. Lipoxygenase (LOX) metabolites including hydroperoxyeicosatetraenoic acids (HpETEs) and hydroxyoctadecadienoic acids (HODEs) are another important example of TRPV1 activators and are discussed below. In addition, the lipid second messenger DAG also activates TRPV1 [60,61] (Figure 3). The binding mode for TRPV1 is quite different from that of TRPA1: Direct binding of DAG was suggested following the observation that it shares the same capsaicin-binding site, while phosphorylation by a downstream kinase was experimentally excluded. Retinoids are known to contribute to axonal outgrowth, neuronal recovery from injuries, differentiation, and stability of the rhodopsin structure. Interestingly, some of these molecular species, including all-*trans*-retinoic acid and 9-*cis*-retinoic acid, can open TRPV1 [62], indicating that its abnormally increased levels in neural disorders may involve a TRPV1-mediated mechanism [63]. Similar to DAG, such retinoids seem to bind to the capsaicin-binding site. Overall, TRPV1 appears to be an extremely polymodal sensor for detection of painful insults.

### 2.3. Inflammatory Pain with TRPA1 and TRPV1

Besides direct detection of physical and chemical harmful insults, participation of sensory TRP channels in the inflammatory signal transduction also occurs in an extensive range. TRPV1 is the best example but evidence for TRPA1 is also increasing. Inflammatory pain mediators including nerve growth factor (NGF), bradykinin, and PGs, through their own G-protein coupled receptor or tropomyosin-receptor-kinase bindings, trigger metabotropic enzymatic cascades that increase either the activity or expression of TRPV1, resulting in increased C-fiber excitation and sensitization [64]. Also, inflamed tissues frequently become acidic, hypertonic, rich in leukotrienes secreted by infiltrated immune cells, and hot owing to increased blood flows. All of these components are able to directly activate TRPV1 [65,66,67]. This is the sensory neuronal (or peripheral) mechanism for detecting inflammatory pain. Similar pain mechanisms explain TRPA1’s role for NGF and bradykinin, and proteases in inflammation [5,6,15,16,68,69], and many of reactive PGs directly activate TRPA1 (see below).

Prolonged excitation of C-fibers can deteriorate inflammatory conditions through a process known as neurogenic inflammation [70], which may be initiated by sustained depolarization due to TRPV1 and/or TRPA1 activation [64]. A subset of C-fibers contains calcitonin gene-related peptide (CGRP) and substance P in their termini, and their excessive excitation caused by the inflammation-TRP axis described above may cause the release of these neuropeptides both in peripheral and central termini. In the periphery, inflamed tissues and infiltrated immune cells can become increasingly stimulated by these peptides and, as a result, release more pro-inflammatory signals. Further, such peptides also have a self-limiting stimulatory potential towards C-fibers [71,72,73,74,75]. In the central synapses, neuropeptide releases contribute to the formation of hyperalgesic synaptic plasticity, which gives rise to chronic pain. Therefore, modulation of TRPA1 and TRPV1 seems to be an important initial step for controlling both inflammation and inflammatory pain states.

## 3. Lipid Peroxidation (LPO) Products Activate TRPA1 and TRPV1

As discussed above, the two major nociceptive TRP ion channels, TRPA1 and TRPV1, act as warning alarms of damaging environments and molecules. TRPA1 and TRPV1 sense these signals, either directly or indirectly, and in turn transduce and relay them to the brain. The resultant pain perception prepares the body for defense. As listed above, the two TRPs are already known to detect a multitude of pathologically generated lipids. Furthermore, several reports have shown that TRPA1 and TRPV1 are also responsive to a certain reactive oxygen species generated during oxidative stress [20].

Oxidative stress often results in LPO. LPO intermediates and end products that acquire covalent reactivity frequently threaten normal biological processes by binding to proteins and biomembranes and changing their structures and functions. This set of interactions raises the following questions: Can it be presumed that reactive LPO lipids are among the set of nociceptive TRP activators, and if so, do TRP function as an alarm for the body to enter a pathologic LPO stage? Surprisingly, the answer seems to be yes, despite the requirement for more systematic accumulation of evidence. Existing cases in support of this possibility are summarized in Table 1. Below, we first briefly introduce the important peroxidized lipids, and then summarize the associated responsiveness of TRPA1 and TRPV1.

**Table 1:** List of the lipid peroxidation (LPO) products that activate sensory transient receptor potential (TRP) channels.

LPO Categories	Oxidized or Peroxidized Lipids	Target TRPs, EC50s, and References
Lipid hydroperoxides (LOX metabolites)	12( *S*)-HpETE 15(*S*)-HpETE 9(*S*)-HODE 13(*S*)-HODE 9(*S*)-HpODE 13(*S*)-HpODE	TRPA1
		HpETEs and HpODEs (not tested)
		9( *S*)-HODE (31.9 μM, [76])
		13( *S*)-HODE (11.6 μM, [76])
		TRPV1
		12( *S*)-HpETE (8 μM, [77])
		15( *S*)-HpETE (8.7 μM, [77])
		9( *S*)-HODE (<0.8 μM, [78,79]; 10–20 μM, [76])
		13( *S*)-HODE (0.8 μM, [79]; 27.5 μM, [76])
		Cf. related HODE metabolites
		9( *S*)-oxoODE (>0.8 μM, [78,79])
		13( *S*)-oxoODE (>0.8 μM, [78,79])
		HpODEs
		Not tested
Isoprostanes	8-iso PGA_1_ 8-iso PGA_2_ J-series isoprostanes	TRPA1
		8-iso PGA_2_ (22.4 μM (in murine DRG (mDRG)), [80])
		Cf. related cyclopentanone PGs:
		PGA_1_ (15.1 μM {mDRG}, [80])
		PGA_2_ (24 μM {mDRG}, [80])
		∆12-PGJ_2_ (10–100 μM, [81])
		15d-PGJ_2_ (5.6 μM, [82]; 1.2 μM, [83]; 8.9 μM {mDRG}, [80])
		TRPV1
		8-iso PGA_2_ (no effect {mDRG}, [80])
		8-iso PGA_1_ & J-series isoprostanes
		Not tested
Acrolein and its analogs	Acrolein Crotonaldehyde Pentenal Hexenal	TRPA1
		Acrolein (5 μM, [6]; 0.8 μM, [84])
		Crotonaldehyde (23 μM, [84])
		Pentenal (5 μM, [6]; ~20 μM, [26])
		Hexenal (~10 μM, [26])
		TRPV1
		Acrolein (no effect, [84])
		Crontonaldehyde (no effect, [84])
		Pentenal (no effect, [26]; excites rodent trigeminal neurons [85])
		Hexenal (no effect, [26])
4-HNE and related alkenals	4-HNE 4-HHE 4-ONE 4-HDDE 4-HPNE 4-HPHE	TRPA1
		4-HNE (13 μM, [26]; 27 μM, [86]; 19.9 μM, [82]; 10 μM, [87])
		4-HHE (38.9 μM, [82]; >50 μM, [87])
		4-ONE (1.9 μM, [82]; 1.6 μM, [87])
		TRPV1
		4-HNE (no effect, [26,86])
		4-HHE (not tested)
		4-ONE (≥100 μM, [87])
		4-HDDE/4-HPNE/4-HPHE
		Not tested
α-Oxoaldehydes	Glyoxal Methylglyoxal	TRPA1
		Glyoxal (no effect, [88])
		Methylglyoxal (0.59 μM (β-cell), [89]; 744 μM, [90]; 341.1 μM, [88])
		TRPV1
		Glyoxal (not tested)
		Methylglyoxal (no effect, [88,90])
Components of oxidized LDLs	Hexanal Pentanal LPC	TRPA1
		Pentanal/hexanal (not tested)
		TRPV1
		Pentanal/hexanal (not tested)
		LPC (not tested)
		Pentanal on native neurons
		An unknown target (1–10 mM? on rat trigeminal neurons, [85])
		LPC
		TRPM8 (~10 μM, [91])
		Cf. related substances
		Formaldehyde (200–400 μM, [26,27])
		Acetaldehyde (76.5–1190 μM, [25,26])
Malondialdehyde	Malondialdehyde	Not tested

TRPA1 and TRPV1, namely pain-mediating TRP channels; HpETEs, hydroperoxyeicosatetraenoic acids; HODEs, hydroxyoctadecadienoic acids; HpODEs, hydroperoxyoctadecadienoic acids; 4-HNE, 4-hydroxy-2-nonenal; 4-HHE, 4-hydroxy-2-hexenal; 4-ONE, 4-oxo-2-nonenal; 4-HDDE, 4-Hydroxy-2,6-dodecadienal; 4-HPNE, 4-hydroperoxy-2-nonenal; 4-HPHE, 4-hydroperoxy-2-hexenal; LDL, Low-density lipoprotein; LPC, Lysophosphatidylcholine; PG, prostaglandin.

### 3.1. Lipid Hydroperoxides (LOX Metabolites) on TRPV1

Among cellular lipid components, PUFAs are relatively vulnerable to the formation of lipid radicals. During oxygen exposure, PUFAs are converted into lipid hydroperoxides either enzymatically (by LOXs) or non-enzymatically. Since diverse PUFAs exist in our body and different hydroperoxide stereomers can be generated depending on the double bond locations, as many as ~150 different lipid hydroperoxides are known to possibly occur in tissues [92]. Derivatives originating from arachidonic acid and linoleic acid are the best examples to examine with respect to TRP channel modulations. Their hydroperoxidation produces HpETEs and hydroperoxyoctadecadienoic acids (HpODEs) (Figure 3). These primary compounds are chemically unstable and later decompose to more stable but still reactive secondary LPO compounds (4-hydroxy-2-nonenal (4-HNE), 4-oxo-2-nonenal (4-ONE), *etc.*). HpETEs can also be metabolized into hydroxy derivatives (hyroxyeicosatetraenoic acids: HETEs) by the glutathione (GSH)-utilizing antioxidant enzyme glutathione peroxidase [93], indicating that it escapes the LPO process. However, binding of some LPO products themselves with this enzyme reduces the enzyme activity, which may lead to LPO accumulation [94]. Hydroxy derivatives (hyroxyeicosatetraenoic acids: HODE) can also form from HpODE, but they seem to have the potential to generate secondary LPO products by forming hydroperoxides with a different carbon atom (hydroxyhydroperoxides) [95].

HpETEs have been shown to activate TRPV1, and HODEs activate both TRPV1 and TRPA1. 12(*S*)-HpETE (a 12-LOX product) and 15(*S*)-HpETE (a 15-LOX product) seem to be relatively more potent for TRPV1 activation than further hydroxyl metabolites (HETEs and leukotrienes) [77]. Among HODEs, 9(S)-HODE (a 5-LOX product) and 13(*S*)-HODE (a 15-LOX product) are both potent TRPV1 activators with several hundred nanomolar EC50s (Table 1). These potencies appear to be the highest among those of endogenous TRPV1 activators identified to date; however, the consistency and even specificity of HODEs has recently come into question, with two research groups suggesting that HODEs may activate both TRPV1 and TRPA1 at tens of micromolar ranges [76,96]. It also remains unclear whether more reactive precursors, HpODEs, are also able to affect these nociceptive TRP activities.

TRP studies on lipid hydroperoxides and its hydroxyl metabolites have not directly been succeeded by further explorations of related LPO ligands. Rather, researchers have focused primarily on different upstream pathways such as pro-inflammatory and thermosensing mechanisms. The former is that a pro-inflammatory mediator bradykinin may utilize contiguous PUFA availability by activating PLA2 and LOX during its Gq-coupled signaling cascade, resulting in TRPV1 activation and subsequent C-fiber excitation [77,97,98]. The latter is based on the argument that thermal activation of TRPV1 may not be a direct one, but may depend on a thermoresponsive *de novo* generation of specific activators [78,79]. This signal transduction hypothesis has been challenged by the intrinsic hypothesis that the protein structure of specific protein domains or of the entire TRP protein may be intrinsically altered in response to temperature changes, which in turn may allosterically alter pore gating [99,100,101,102], although this is beyond the scope of this review. Future studies are needed to clarify which diseases caused by LPO processes are particularly vulnerable to the presence of lipid hydroperoxides and whether TRP activation contributes to recognition of disease states.

TRP activation by these lipid hydroperoxides is unlikely to be due to covalent modifications such as Michael addition or Schiff base formation because of the absence of reactive α,β-unsaturated carbonyl carbons in their lipid structures (see next section) (Figure 3). On the other hand, a typical non-covalent lock-and-key model and subsequent allosteric gating may explain the activation mechanism. A strong candidate site for non-covalent binding with lipidergic ligands comprising the linker between the 4th and 5th transmembrane domains (TM4–TM5 linker or S4–S5 linker) was recently suggested [103]. The S4–S5 linker in a voltage-gated ion channel is known to transform the physical movement of the S4 region which is sensitive to depolarization into pore gating. Lipid hydroperoxides may mimic this allosteric mechanism via its binding to S4–S5 linker of TRPV1. It remains to be seen whether this theory commonly explains interactions with other non-covalent LPO products.

### 3.2. Isoprostanes on TRPA1

**Figure 4:**
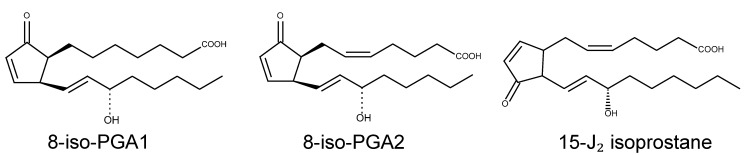
Isoprostanes.

PUFAs can generate another category of LPO products known as isoprostanes [104,105]. Independent of the cyclooxygenase (COX) pathway, non-enzymatic and free radical-mediated multi-step oxygenations of PUFAs produce PG-like substances. A PG ring is formed in the middle of the aliphatic structure and through a further dehydration step, some subtypes (A, B, and J series) acquire one or two α,β-unsaturated carbons inside and/or near the PG ring. As a result, the PG ring is typically a cyclopentenone one. Among the reactive cyclopentenone isoprostanes, 8-iso PGA_2_ was shown to selectively activate TRPA1 [80] (Figure 4). Other reactive isoprostanes including 8-iso PGA_1_ and J-series have not yet been evaluated for TRP examination. Interestingly, although none are isoprostanes, several COX-produced cyclopentenone PGs also exhibit similar potencies on TRPA1 activation, namely, 15-deoxy-Δ12, 14-PGJ_2_, and Δ12-PGJ_2_, PGA_1_, and PGA_2_, [80,81,82,83]. Irrespective of whether they are isoprostanes or COX-produced PGs, TRPA1 activators commonly have α,β-unsaturated carbons and seem to follow the covalent binding principle that targets cytoplasmic *N*-terminal nucleophilic amino acids on TRPA1 protein (see next paragraph). On the other hand, TRPV1 is inert to all the reactive isoprostanes and PGs described above that have been shown to activate TRPA1. Thus, these direct isoprostanes/PGs detections appear to be only in charge of TRPA1.

The covalent binding principle seems to hold true with respect to the TRPA1 activation mechanism not only for the cyclopentenone isoprostanes/PGs described in this section, but also for the reactive LPO aldehydes listed below [7,106]. As mentioned previously, the critical sites for TRPA1 covalent binding are located in the cytoplasmic side (*N*-terminal nucleophilic lysine and cysteine residues). When LPO products containing α,β-unsaturated carbons are present in the cytosol, *N*-terminal lysine (ε-amino group), cysteine (sulfhydryl group), or histidine (imidazolyl group) of TRPA1 nucleophilically attacks those carbons to form covalent bonds (Michael addition). Furthermore, carbonyl-containing electrophiles can react with lysine residues or free *N*-terminal amino groups to form Schiff base products. In the case of some LPO products including 4-HNE, 4-ONE, hexanal, and methylglyoxal, Schiff base formation with the amines of lysine residues of a target protein is known to occur, and this reaction appears to be less reversible than Michael addition [107,108,109]. Therefore, it is worth examining the possibility of Schiff base adduct formation as well as Michael adduct formation when TRPA1 activity is aberrantly sustained. However, not all of the susceptible amino acids on the *N*-terminal appear to readily convert binding into pore gating. Hinman *et al.* identified the three most critical cysteine residues and one lysine residue (C619, C639, C663, and L708 in the human ortholog) in this regard [106]. Two more cysteines (C415 and C422 in the mouse ortholog) were reported in a study performed by Macpherson *et al.* [7]. Mechanistic details remain unclear on how allosterically and structurally *N*-terminal covalent modification affect pore gating.

### 3.3. Acrolein on TRPA1

**Figure 5:**
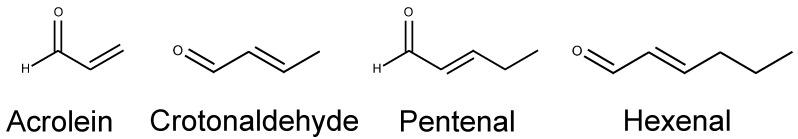
Acrolein and its analogs.

Acrolein is an environmental pollutant created by combustion of organic matters and is also generated endogenously in metal-catalyzed oxidation of PUFAs such as linoleic acid, arachidonic acid, eicosapentaenoic acid, and docosahexaenoic acid [110]. Acrolein is also produced from polyamines, which are TRPV1 activators, via the activity of amine oxidase [111,112]. Acrolein is among the most reactive LPO products (~100 times more reactive than 4-HNE in terms of electrophilicity). This high reactivity of acrolein might occur in its covalent interaction with TRPA1 and result in a relatively high potency for TRPA1 activation (EC50 = 0.8 µM) (Table 1). However, the EC50 for activation of TRPA1 by acrolein was initially reported as 5 µM [6], and thus additional studies will be necessary to confirm its potency. The same study also reported that TRPV1 resisted acrolein treatment. Moreover, many different forms of protein adducts have been reported such as propanal adduct, cyclized adduct, *N^ε^*-(3-formyl-3,4-dehydropiperidino)-lysine adduct, *etc.* regarding acrolein reactions [113]. Thus, it may be interesting to determine which form bound to which susceptible amino acid is responsible for the majority of acrolein’s maximal TRPA1 activation. Crotonaldehyde, pentenal, and hexenal are acrolein analogs with a longer carbon chain, and also specifically open TRPA1 but have a mild potency (EC50 values = 23, 5–20, and ~20 µM respectively) [6,26,84] (Table 1) (Figure 5).

Despite not being related to LPO, acrolein is also a toxic end-metabolite of alkylating agents for chemotherapy including cyclophosphamide and ifosfamide [114,115,116,117]. Acrolein accumulation leads to severe adverse effects such as hemorrhagic cystitis with pain, which may be caused by TRPA1 activation and associated neurogenic inflammation (see above). Thus, mechanistic control of the acrolein-TRPA1 interaction axis may help to attenuate the adverse effects.

### 3.4. 4-HNE and Related 2-Alkenals on TRPA1

**Figure 6:**
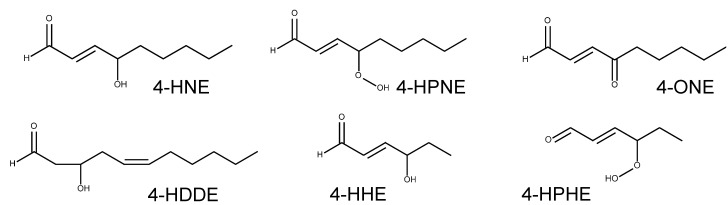
Hydroxy-2-nonenal (HNE) and related alkenals.

Lipid hydroperoxides generated from PUFAs may undergo complex non-enzymatic LPO processes, which are in general constituted by alkoxyl radical formation, further oxygenation, and fragmentation [118]. Consequently, 2-alkenals with diverse carbon lengths are formed, among which the best examples in TRP channel studies have been 4-HNE, 4-ONE, and 4-hydroxy-2-hexenal (4-HHE) (Figure 6). 4-HNE and 4-ONE are generated from ω-6 PUFAs including arachidonic acid and linoleic acid while 4-HHE is formed from ω-3 PUFAs such as docoshexaenoic acid, eicosapentaenoic acid, and linolenic acid [119]. Compared to lipid hydroperoxides, these fragmented end products are more chemically stable and diffusible through cell membranes and thus can travel to locations outside of those where they were originally produced [120,121,122]. GSH conjugation (which is also a Michael addition) seems to be the major step for 2-alkenal scavenging, and this reaction can be both spontaneous and catalyzed by glutathione-*S*-transferases [118]. However, because this reaction is reversible, alkenals occasionally travel as far as a GSH-bound form, dissociate again, and then covalently bind to local proteins, which may contribute to spreading of oxidative disease regions [118]. Physiological 4-HNE levels are variable (0.3–100 µM, the plasma level is generally lower than tissue levels). Under disease states during oxidative insults, two to more than 100-fold increases have been detected (5 µM–5 mM) [120]. 4-ONE was once reported to achieve a comparable level with that of 4-HNE *in vitro* [123].

Activation of TRPA1, but not TRPV1 was exerted by relevant 4-HNE concentrations. The EC50 values for TRPA1 activation range from 10 to 30 µM in four different studies [26,82,86,87]. 4-ONE is a more potent TRPA1 activator. Its EC50s were 1.6 and 1.9 µM in two independent studies, which indicates that 4-ONE has an approximately 10 times-greater potency [82,87]. This is consistent with the differences in their electrophilic potentials. Owing to their structural differences (4-ONE has a carbonyl moiety in the same place as the hydroxyl moiety of 4-HNE), 4-ONE’s electrophilic reactivity appears to be dozens of times greater than that of 4-HNE, which may explain the more reactive protein modification or cross-linking indices of 4-ONE [124,125]. Acrolein is similarly strong with respect to TRPA1 activation as described above. It is notable that 4-ONE and acrolein might react with TRPA1 in different modes of covalent modification since it was reported that acrolein is a better carbonyl incorporator but that 4-ONE is a better cross-linker [121]. Modest TRPV1 activation by 4-ONE was also observed in the hundred-micromolar range [87]. At such concentrations, which can be reached under pathological conditions, TRPV1 might also take part in raising the alarm to elevated levels of 4-HNE. 4-HHE exhibits only a mild TRPA1 activation effect (Table 1). When ω-6 PUFAs decompose to 4-HNE and 4-ONE, both substances can be generated through the same intermediate, 4-hydroperoxy-2-nonenal (4-HPNE). Likewise, 4-hydroperoxy-2-hexenal (4-HPHE) is an intermediate for 4-HHE. However, these two compounds have yet to be tested for TRP channel modulation. 4-Hydroxy-2,6-dodecadienal (4-HDDE), produced only dependent on 12-LOX action on arachidonic acid and known as a potent agonist for peroxisome proliferator-activated receptor γ, also remains to be examined [126].

### 3.5. Methylglyoxal on TRPA1

**Figure 7:**
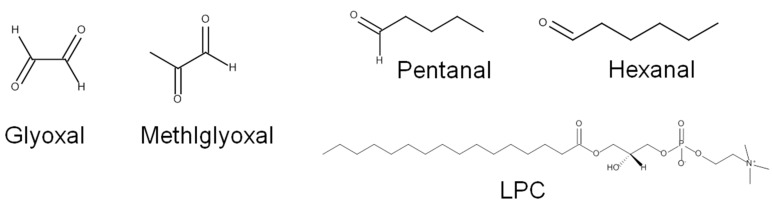
α-Oxoaldehydes, alkanals, and LPC.

α-Oxoaldehydes such as glyoxal and methylglyoxal are formed not only from degradation of sugars or glycated protein in hyperglycemic conditions, but also from LPO [127,128,129] (Figure 7). Surprisingly, earlier than other lipids describe above, glyoxal was reported to covalently modify an ion channel function in the nervous system [130]. At the time, its utility was viewed as more practical in that experimental covalent binding of certain reagents to particular arginine groups might be useful for enhancing our understanding of the structure-function relationship of ion channel proteins. Thus, the implications of glyoxal in biological systems where it occurs naturally were not considered, perhaps also because evidence for natural interactions was still accumulating. In fact, pioneering studies performed even earlier demonstrated that α-oxoaldehydes could be formed endogenously in bio-organisms [131,132,133]. In the late 60s and early 70s, these substances began to be considered as toxic byproducts generated from glycolysis and covalent protein modifiers [134,135]. Finally in the 90s, studies suggested that LPO, including autooxidation of PUFAs, might produce these oxoaldehydes [136,137]. A similar mechanism (Michael addition or Schiff base formation) explains the covalent modifications of the critical amino acids (lysine, histidine, arginine, and cysteine), and over a period of days, an additional chemical rearrangement may form Amadori products (keto-forms), which can proceed to further reactions to generate harmful end products (advanced glycation end products: AGEs) on a longer term basis [138].

Methylglyoxal, has been tested in TRP activation multiple times. Methylglyoxal was initially shown to cause membrane depolarization in pancreatic β cells at relatively low concentrations (EC50 = 0.59 μM), and also cause insulin secretion [89]. Both of these functions are ablated by RNA interference-mediated TRPA1 depletion or pharmacological inhibition. A month after this observation was reported, an independent group demonstrated that methylglyoxal activation of TRPA1 is mediated by a direct covalent reaction with critical cysteine residues, but not with lysine. They also detected elevation of nociceptive firing and CGRP release in sensory nerves [90]. Another group confirmed this activation in the same year and further showed that TRPA1 was not responsive to glyoxal application [88]. The latter two studies obtained EC50 values in the order of hundreds of micromolars (Table 1), very different from the initially reported values, which may have been due to tissue-specific supporting components, although this remains unclear. Methylglyoxal seems to be specific for TRPA1 among sensory TRPs, since TRPV1 activity is unchanged in its presence [88,90]. Besides TRPs, other ion channels including Nav1.8 voltage-gated Na^+^ channel and Kir6.1/SUR2B ATP-sensitive K^+^ channels have also recently been suggested to respond to methylglyoxal modifications [139,140], but only TRPA1 has demonstrated to be essential with respect to pain detection [141].

### 3.6. LDL-Related Lipids on TRPA1

Low-density lipoprotein (LDL) comprises a complex of numerous lipid species and proteins. During LPO, lipid components are oxidized or peroxidized first and the resulting components may in turn covalently modify protein components including apolipoprotein B. Alternatively, LPO products formed outside LDL under widely oxidative conditions, may also modify those proteins [118], and in either state, those are referred to as oxidized LDLs (oxLDLs). The oxLDLs may affect TRP ion channel activities not in a whole form, but in a manner whereby particular oxidized lipid components act on TRP channel proteins. However, major components, such as oxidized cholesterols and oxidized phospholipids remain to be experimentally examined. The effects of some of the lipid components on TRPs have independently been evaluated in the context of identifying TRP channel ligands and endogenous lipidergic activators.

Certain alkanals such as pentanal and hexanal have been shown to be increased during LDL peroxidation [142,143] (Figure 7). Only pentanal was tested at millimolar ranges, and it was found to induce intracellular Ca^2+^ increases in nociceptive populations of rodent trigeminal neurons [85]. This study proposed that pentanal might interact with an unknown receptor molecule, which is unlikely TRPV1 because only a small subset of capsaicin responders respond to pentanal. The possibility of TRPA1 participation was excluded in an independent study [26]. Interestingly, despite not being among the LPO products, the simplest alkanals including formaldehyde and acetaldehyde have been shown to specifically activate TRPA1 with a higher potency than observed in the pentanal study above [25,26,27]. The greater potencies for TRPA1 activation by alkenals including pentenal and hexenal as described above in the acrolein section, and also by those of alkenals with other carbon chain lengths such as butenal, heptenal, and octenal [26], illustrate that the presence of an α,β-unsaturated carbonyl moiety is of fundamental importance for TRPA1 activation. Lysophosphatidylcholine (LPC) may be enzymatically released from oxidized phospholipids in oxLDLs [118] (Figure 7). LPC was also shown to activate and sensitize melastatin subtype 8 of TRP channels, which is another receptor for cold temperatures and menthol [91,144,145]. *In vivo* animal injection of LPC leads to cold hypersensitivity, and thus LPC is among nociceptive endogenous molecules that exert their effect via TRPM8 activation [145]. It remains to be determined whether these individual lines of evidence support the notion that increased levels of oxLDL during LPO causes nociception by stimulating TRP channels. Other possibilities can also be considered. For example, oxLDLs might modulate TRP channel functions via signal transductions. Consistent with this possibility, two studies have shown that activities of Ca^2+^-activated K^+^ channels and L-type Ca^2+^ channels are enhanced via lectin-like-oxLDL-receptor-1 signaling and via increased mitochondrial reactive oxygen production, respectively, although these findings were not related to a sensory mechanism [146,147].

## 4. Discussions and Perspectives

Although this review tried to collect and reconstitute the information on the interactions between peroxidized lipids and TRPA1/TRPV1 from a consistent angle, few experimental studies have thoroughly evaluated TRP activity states in the context of LPO severity. Nonetheless, the existing data suggests that TRPA1 is likely a dominating detector for LPO products and that TRPV1 also plays a role in this process. From this viewpoint concerning the LPO process, from the birth of primary hydroperoxy lipids with a relatively longer carbon chain such as HpETEs through fragmented end products including acrolein and HNE, detection in the relatively early stage of this process might be in charge of TRPV1 whereas those for the later (and more severe and reactive, too) stage might instead involve TRPA1. To evaluate this possibility, thorough studies aimed at filling in the blanks for TRP effects of many other untested LPO products will be necessary. Several of these lipids whose targets remain to be identified are also listed in Table 1 (HPODEs, 8-iso PGA_1_, HDDE, *etc.*). Malondialdehyde might be interesting since it is present in larger amounts (about 80-fold) than HNEs in the body, but is a relatively weak electrophile and seldom targets cysteine, different from other reactive secondary products. Lipofuscin is a granular pigment progressively formed in various tissues during aging [148]. The accumulation of lipofuscin is closely associated with diseases related to aging such as macular degeneration and Alzheimer disease. It is a collection of multi-type LPO products including malondialdehyde and other lipids described above, as well as their protein adducts, and exposure to extreme oxidative stress may exacerbate lipofuscin accumulation. Lipofuscin is not presently considered to be just a waste cargo for degraded lipids, but is also thought to be a factor affecting disease development by communicating with cellular pathological molecules. In this regard, the possible correlation with functions and expression levels of interacting TRP channels and causative sensory states could be explored. During LPO, isoprostanes can further be rearranged into γ-ketoaldehydes, and isoketals that are still sufficiently reactive to form covalent adducts on proteins or lipids [149], while TRP interactions and related outcomes remain unexplored.

Changes in plasma membrane fluidity or thickness caused by altered lipid architecture may affect an ion channel protein structure and eventually its gating properties [3]. For example, the thermal sensitivities of TRPV1 and TRPM8 are altered when these channels are located in cholesterol-rich regions [150,151,152]. Since some LPO products not only bind to proteins but also membrane lipids, this might affect the bilayer architecture. HNEs are among the LPO products known to modify phosphatidyl ethanolamine [153]. It remains elusive whether such a membrane effect can indirectly contribute to TRPA1 activation. In addition to such microscopic chemicostructural changes, LPO processes in the plasma membrane lead to bleb formation, possibly via covalent modifications of the underlying cytoskeletons [154]. As a result, sustained increases in macroscopic bilayer tension may chronically elevate sensitivities of TRPA1 and TRPV1, both of which have been shown to be activated by an increase in membrane tension [15,16,18,50].

TRPA1 and TRPV1 are central sensory alarms for acute pain to avoid potentially harmful damage. In a chronic disease stage, however, those are often turned into important aggravating factors amplifying neurogenic inflammation (see above). For LPO processes as well, it should be cautiously considered whether tuning on those two alert signals simply indicates a DEFCON-type warning or contributes to a deteriorating state of war that has already begun. Indeed, TRPA1-mediated neuronal excitation by 4-HNE, isoprostanes, acrolein, and crotonaldehyde causes the release of neuropeptides such as CGRP or substance P, which are key amplifiers of neurogenic inflammation signaling in the periphery and potentiators for nociceptive synaptic strength in the central synapse [80,84,86,90]. Crotonaldehyde is unlikely to be a TRPV1 activator [84], but its hour exposure elevates TRPV1 expression, which may in turn promote inflammatory pathways [155]. Furthermore, in addition to TRP channels, LPO products may modify other components in pathological pain pathways. For example, activation of the mitogen-activated protein kinases pathway is crucial for developing chronic pathological pain [156]. HNEs and ONEs have also been shown to strongly facilitate this pathway [157,158]. Cortical spreading depression, which is considered to be a mechanism for migraine aura, appears to be associated with increased levels of reactive oxygen species and LPO products, and their interactions with TRPA1 and the resulting CGRP release may exacerbate migraine pathology [159]. If TRPA1/TRPV1 are important players for pathological pain development during oxidative stress, it might be conceivable that LPO levels in the serum or urine, can be utilized as indices for quantitatively determining how pain shifts towards chronic and pathological states. Similar concepts are becoming more and more recognized in other diseases including colon cancer and atherosclerosis [160,161,162,163].

The LPO-TRP axis might also function in other pathologies in addition to pain induction depending on the locations of TRP channels. For example, TRPA1 is abundant in auditory hair cells [164] and LPO products including 4-HNE serve an important role in hair cell degeneration and hearing loss following noise exposure [165]. It will be interesting to see whether excessive activation of TRPA1 by LPO products contributes to hearing loss and if TRPA1 is a viable therapeutic target for hair cell protection.

The sensory nerve terminal innervating tegumental tissues may readily be exposed to oxidative attacks originating from the environment, which is one of the reasons that the nerve terminals should have detector molecules such as TRPA1 and TRPV1 as mentioned above. Moreover, the nerve itself is fundamentally a tissue with a high probability of generating more oxidative stress due to its higher oxygen demand than other tissues. Thus, sensory nerves may be among the tissues that need to tightly maintain a homeostatic balance of the oxidative state by equipping it with protection systems, for example, glutathione-*S*-transferases and alkenal oxidoreductases; however, this possibility has not been thoroughly studied [166]. In light of the fact that exogenous treatment of glutathione is protective of shifting TRPA1 activity to a normal stage in the context of exposure to LPO products [84,87], it is challenging to see how considerably neuronal enzymatic systems prevent possible oversensitizations of TRP channels, and how much of an effect this may have in terms of *in vivo* EC50 values reflecting their mechanism and *in vitro* EC50 values that have previously been observed under enzyme-free conditions.

Collectively, we tried to reconstitute LPO information from scattered TRPA1/TRPV1 ligand studies performed under a diverse set of interests. There are numerous areas that need to be researched in order to form a conclusive determination of how interactions are mechanically constructed, between the LPO network and the sensory network in the nervous system. Future findings will contribute to precisely defining the sensory roles of TRP channels in LPO-associated diseases or LPO protection, and help to identify the ways in which we can utilize LPO and TRP indices to improve human health and discover novel approaches against oxidative pathologies.
